# Storage and in-use stability of an excipient enhanced growth (EEG) synthetic lung surfactant powder formulation

**DOI:** 10.1080/03639045.2025.2508845

**Published:** 2025-05-26

**Authors:** Mohammad A. M. Momin, Connor Howe, Worth Longest, Michael Hindle

**Affiliations:** a Department of Pharmaceutics, Virginia Commonwealth University, Richmond, VA, USA; b Department of Mechanical & Nuclear Engineering, Virginia Commonwealth University, Richmond, VA, USA

**Keywords:** Excipient enhanced growth, lung surfactant, powder aerosol, formulation, stability

## Abstract

**Objective:**

To evaluate the storage and in-use stability of a novel synthetic lung surfactant (SLS) excipient enhanced growth (EEG) powder formulation.

**Significance:**

Aerosol delivery of surfactant formulations could address limitations of current instilled surfactant replacement therapy for neonatal respiratory distress syndrome. A stable surfactant powder formulation is essential for this approach.

**Methods:**

SLS-EEG powder was spray-dried from a formulation of dipalmitoyl phosphatidylcholine (DPPC), surfactant protein B peptide mimic (B-YL), mannitol, sodium chloride, and l-leucine. Powders were filled into hydroxypropyl methylcellulose (HPMC) capsules and stored in aluminum-aluminum blisters at 25 °C, 5 °C and −20 °C (all ± 2 °C) and 40 ± 5% relative humidity (RH) for 6 months. Physicochemical and aerosol properties were assessed post-spray drying and post-storage. In-use stability was assessed by exposing powders to 22 °C/45% RH for 30 and 120 min and 30 °C/75% RH for 120 min before dry powder inhaler (DPI) actuation.

**Results:**

DPPC content remained stable for 6 months at all storage temperatures. Powder morphology and particle size were unchanged at 5 °C and −20 °C, but altered at 25 °C. Solid-state stability and surface activity were unaffected. Emitted doses remained high (>95%) after 3 months using an infant air-jet DPI, though *in vitro* lung deposition decreased from ~50% to ~40% and ~30% at 3 and 6 months. In-use exposure of the formulation in device to 22 °C/45% RH caused no lung deposition changes, but it declined at 30 °C/75% RH (~40% vs. ~50%).

**Conclusions:**

A synthetic lung surfactant EEG powder formulation with physicochemical stability and acceptable aerosol performance up to 6 months storage has been successfully produced.

## Introduction

Instilled surfactant replacement therapy (SRT) is the first-line treatment for neonatal respiratory distress syndrome (NRDS), the most common lung disease in preterm neonates [1,2]. However, non-uniform distribution of the surfactant in the lung airways, respiratory related side effects arising from intratracheal instillation of a high liquid volume through the endotracheal tube and the requirement for multiple doses of surfactant are the most common problems in liquid bolus instillation of SRT [3–5].

Surfactant delivery as aerosol formulations has been envisioned for a number of decades as a method to effectively treat NRDS without the side effects and risks associated with instilled SRT through an endotracheal tube and other less invasive liquid bolus installation techniques [6–11]. Nebulization has been broadly studied to generate and deliver surfactant aerosols to treat NRDS [11–15]. However nebulized delivery of surfactant aerosols has some limitations including the prolonged delivery time and variable clinical efficacy [14,16]. Dry powder aerosolized surfactant therapy showed promising preliminary results in small human subject trial over several decades ago [8] and could be an alternative to overcome the problems associated with nebulization. Moreover, dry powder aerosols offer a number of advantages over liquid nebulization including product stability, higher delivery efficiency and less susceptibility to microbial growth [17–19]. Despite these advantages, dry powder aerosol delivery to infants is not often considered since most of the commercial dry powder aerosol products are designed for adults [19–22]. Efficient delivery of dry powder aerosols to neonates faces a number of challenges due to their low tidal volumes and the different inhalation profiles compared to the adult population [23]. Generally, the inhalable micrometer-sized dry powder aerosol particles are difficult to disperse into their primary particles, and it is more challenging for neonatal delivery when powder needs to be dispersed using a very low air actuation volume of ~10 ml.

We have developed highly dispersible spray-dried formulations using our excipient enhanced growth (EEG) formulation technology and a positive-pressure infant air-jet DPI platform for nose-to-lung administration of EEG formulations to infants that are ideal for aerosol administration to infants with small actuation air volumes and low air flow rates [24–27]. We reported the development of a spray-dried powder aerosol formulation produced from a commercially available endogenous replacement surfactant product, Survanta^®^ intratracheal suspension, using the EEG approach. The aerosol performance of the Survanta EEG formulation in a novel low air volume dry powder inhaler (DPI) was also described previously [28,29]. By implementing the newly developed positive-pressure infant air-jet DPI platform, we also recently explored the effects of different flow rates and DPI designs on the nose-to-lung delivery of the Survanta EEG powder formulation through a preterm NT model. This study concluded that, the air-jet DPI platform (with three inlet air-jet D5 device) is capable of high-efficiency aerosolization (~95% emitted dose) of a 10 mg Survanta EEG powder by using 10 ml air actuation volumes at a flow rate of 4 LPM [30]. The positive-pressure infant air-jet DPI platform has four main components: the air source, the aerosolization engine, a pressure monitoring and control unit and the nasal interface. The air source is responsible for providing the aerosolization energy to the air-jet DPI, the aerosolization engine is responsible for aerosolization of the powder, the pressure monitoring and control unit ensures patient safety with a pop-off valve and the nasal interface forms an airtight seal with an infant’s nostril to deliver the powder aerosol to infant lungs. As the air source is actuated, high speed air jets pass through the aerosolization chamber of the air-jet DPI preloaded with powder facilitating powder aerosolization. The aerosol then passes through the nasal interface and to the infant [30]. To accommodate the delicate nasal passages of neonates, in our DPI platform we intend to use soft, atraumatic nasal interfaces with low inspiratory flow requirements which aligns with the neonatal respiratory physiology in combination with airflow rates and air volumes appropriate for the premature infant population. Spray drying techniques employed to produce the Survanta EEG formulation are becoming increasingly popular methods of particle engineering that are easily scalable [31,32]. However, spray-dried particles are mostly amorphous in nature which remains a significant challenge affecting the physicochemical stability of the powder particles [33]. Recently, we reported a preliminary study on the storage stability at −20 °C and 0% RH of the Survanta EEG spray-dried formulation [34]. The preliminary storage stability study showed physicochemical stability of the powder with minimal changes in crystallinity over 18 months storage and no changes in the emitted mass and particle size.

As we further develop the powder surfactant aerosol EEG platform, our formulation development has evolved from an animal-derived Survanta-EEG formulation to a synthetic lung surfactant (SLS)-EEG formulation. The spray-dried (SD) SLS-EEG formulation contains dipalmitoylphosphatidylcholine (DPPC), a surfactant protein B peptide mimic (B-YL), hygroscopic excipients (mannitol and sodium chloride) and dispersion enhancer (l-leucine) [35,36]. Our choice to transition from an animal derived formulation (Survanta-EEG) to a pharmaceutically synthetic formulation (SLS-EEG) was based on similar and in some cases improved aerosol performance, improved raw material availability and significantly reduced current and future costs (~10 to 100-fold lower). The current study aims to evaluate the storage and in-use stability of a novel SLS-EEG formulation. For storage stability, the aerosol performance and physicochemical properties of the capsule/blister packaged SLS-EEG formulation, were evaluated after 3- and 6-months storage at 25 ± 2 °C and 40 ± 5% RH (ambient), 5 ± 2 °C and 40 ± 5% RH (fridge) and −20 ± 2 °C and 40 ± 5% RH (freezer). For in-use stability the aerosol performance was evaluated by environmental exposure of the powder formulation for 30 min and 120 min at 22 °C and 45% RH and 120 min at 30 °C and 75% RH in the DPI prior to actuation.

## Materials and methods

### Materials

Synthetic DPPC with a purity of >99% and surfactant protein B (SP-B) peptide mimic (B-YL) with a purity of >95% were obtained from Avanti Polar Lipids, Inc. (Alabaster, AL) and CSBio (Menlo Park, CA), respectively [35,36]. L-Leucine (analytical reagent grade) was purchased from Sigma-Aldrich Chemical Co. (St Louis, MO). Analytical reagent grade sodium chloride, D-mannitol, ammonium formate and HPLC grade ethanol and methanol were procured from Fisher Scientific Co. (Hanover Park, IL).

### Preparation of the SLS-EEG formulation

In this study three separate batches of SLS-EEG powder were spray-dried. We first prepared the feed dispersions by combining all the formulation components (DPPC:25% w/w, B-YL:3% w/w, mannitol:42% w/w, sodium chloride:10% w/w and L-leucine:20% w/w) in ethanol-water (5:95% v/v) to make final solid contents of 0.125% w/v [28,36]. Then we spray-dried the liquid feed in a Nano spray dryer (Büchi Labortechnik AG, Switzerland) with an open mode configuration and stated operating conditions [28,35]. The set operating conditions were: inlet gas flow 120 L/min, inlet temperature 70 °C, pump speed 5% and spray intensity 80%. The resulted outlet temperature at these set operating conditions was 35–38 °C [28,35]. The measured spray rate was determined as the amount of feed solution (mL) sprayed in a specific amount of time (min).

### Powder collection, initial evaluation and storage

The spray-dried formulations were collected from the electrostatic precipitator into glass vials and stored sealed at 0% RH and −20 °C. After initial evaluation of the powder batches (130–160 mg batches), they were combined together and ~10 mg filled in each size 0 HPMC capsules (Qualicaps Europe, Madrid, Spain), packaged in aluminum-aluminum blister strips with NFA-003 cold-formable aluminum plastic laminates and PFH-092 blister lidding (Amcor Flexibles, KY, USA) using blister packing machine (Sepha EZ Blister, Belfast, Ireland) and stored at 25 ± 2 °C and 40 ± 5% RH (ambient), 5 ± 2 °C and 40 ± 5% RH (fridge) and −20 ± 2 °C and 40 ± 5% RH (freezer) for a 6 month stability study [36].

### Measurement of powder bulk density

The bulk density of each spray-dried SLS-EEG formulation batch was determined using a 0.1 ml stainless steel container (Micromeritics, Georgia, USA). Briefly, the container was gently filled with each batch of formulation and recorded the mass. The mass was then divided by the known volume (0.1 ml) of the container to calculate the powder bulk density.

### Evaluation of content uniformity

The DPPC content in the formulation batches (individual and combined) was determined using an LC–MS method [28,35]. Each sample was prepared by dissolving ~1 mg of SD formulation in 50 ml of methanol and quantitatively analyzed for DPPC content. For each formulation sample, triplicate preparations were analyzed. Due to changes in B-YL content during spray drying, its stability was not assessed and only the stability of the DPPC component of the formulation was investigated.

### Evaluation of moisture content

The Pyris 1 thermogravimetric analyzer (TGA) (PerkinElmer, Covina, CA) with TAC 7/DX thermal analysis controller was used to estimate the moisture content of the formulation batches. A small amount of formulation (~5 mg) was loaded in an aluminum pan. Under a nitrogen environment (purging at 40 ml/min) the samples were heated at 10 °C/min from 25 to 100 °C. The percentage of moisture content was determined from the percent of weight loss following an isothermal hold at 100 °C for 45 min.

### Evaluation of particle size distribution

The SD SLS-EEG formulation batches (individual and combined) were evaluated for particle volumetric diameters at initial and after storage at different conditions using the Sympatec HELOS system at 4.0 and 0.5 bar dispersion pressures [28]. About 2–3 mg of SLS-EEG powder was placed in a Sympatec vial and introduced into the ASPIROS sample feeder. At a specified pressure, the powder sample was dispersed into the laser beam using the RODOS/M disperser with an R1 lens [28]. The size measurement was reported as Dv50, along with the percentage of fine particle fractions (<1 μm and <5 μm), calculated using WINDOX 5.0 software. Particle size results at 4 bar are indicative of fully de-aggregated primary particles, while results at 0.5 bar reflect the powder’s dispersibility.

### Evaluation of particle morphology

The morphology of the SD SLS-EEG particles at initial and after storage at different conditions was examined by capturing the images using a scanning electron microscope (SEM) (Carl Zeiss, Oberkochen, Germany) [35]. Approximately 1 mg of the formulation was placed on a metal SEM stub with double-sided adhesive tape using a spatula. Loose powder particles were removed with compressed air, and the samples were coated with a thin layer of gold using an EMS550X sputter coater (Electron Microscopy Sciences, Hatfield, PA) [35]. SEM images were captured at a magnification of 5k.

### Evaluation of solid-state stability

The spray-dried SLS-EEG formulations were evaluated for their solid-state properties at initial and after storage using the Rigaku Miniflex 6 G (Rigaku, Japan) X-ray powder diffractometer (XRPD) with a Cu-Kα radiation source generated at 40 kV and 40 mA [35]. About 3–5 mg of formulation was placed on the center of the XRPD sample holder and then the powder surface was gently smoothed using a glass slide. The samples were scanned at room condition using a continuous mode in the range of 3–50° 2θ at a scan speed of 1.5°/min [35].

### Evaluation of moisture sorption

The water uptake of the freshly prepared SD SLS-EEG formulations was evaluated based on their moisture sorption behavior at different relative humidity (RH) levels using a dynamic vapor sorption (DVS) system (DVS Adventure, Surface Measurement Systems Ltd., UK) [35]. Approximately 5 mg of the formulation was first equilibrated at 0% RH before being subjected to a sorption cycle with gradually increasing RH levels from 0% to 95%, followed by a desorption cycle with decreasing RH from 95% to 0%. At each RH level, the system progressed to the next stage once equilibrium was reached, defined as a rate of mass change (dm/dt) below 0.002%.

### Evaluation of aerosol performance and lung delivery efficiency

The aerosolization performance of the formulation and lung delivery efficiency was evaluated using an infant air-jet DPI connected to a preterm nose-throat (NT) model [37]. In this aerosolization study, the previously reported D5 air-jet DPI device was used [30]. The air-jet DPI was connected to the airway model and actuated with a positive-pressure air source at 4 L/min. An actuation air volume of ~10 ml was used to deliver 10 mg of formulation through the nasal interface and preterm NT model to the outlet tracheal filter. Aerosolization performance was determined based on the device emitted dose, and lung delivery efficiency was determined from the DPPC deposited on the tracheal filter. The DPPC content was determined by dissolving the deposited formulation using appropriate amount of methanol for each deposition site and quantitatively analyzing for the DPPC content by the LC–MS method described previously [28,38]. Emitted dose (ED) was calculated as the mass of DPPC in the loaded dose minus the mass of DPPC remaining in the device divided by the initial loaded mass of DPPC (expressed as a %nominal). The DPPC deposited on each site of the system was calculated as percentage of the DPPC present in the loaded mass of formulation.

### Storage and in-use stability

The physicochemical and aerosolization stability of the SLS-EEG formulation were evaluated after 3 months (3 M) and 6 months storage (6 M) at 25 ± 2 °C and 40 ± 5% RH (ambient), 5 ± 2 °C and 40 ± 5% RH (fridge) and −20 ± 2 °C and 40 ± 5% RH (freezer). For physicochemical stability we characterized for DPPC content, particle size, particle morphology, and XRPD following the methods described above. The aerosolization performance and lung delivery efficiency following storage were also evaluated. In addition, the formulations were also evaluated for in-use stability (aerosol performance) by environmental exposure of the powder formulation. In this study, the formulation was transferred from the capsule into the aerosolization chamber and loaded into the assembled inhaler device and exposed for 30 min and 120 min at 22 °C and 45% RH and 120 min at 30 °C and 75% RH in the DPI prior to actuation.

### Surface activity

The dynamic surface tension of the SLS-EEG formulation initially and after storage was measured using bubble pressure tensiometer (BP100, Krüss Scientific, Hamburg, Germany). Briefly, the SLS-EEG formulations were prepared with a target DPPC concentration of 0.5 mg/mL and dispersed in 5 mM NaCl using a microtip probe sonicator (Qsonico LLC, CT, USA) at an amplitude of 35 Hz for ~2 min. Surface tension measurements of the dispersions were performed with the tensiometer connected to a recirculating water bath (Fisher Scientific, Waltham, MA) that allowed for temperature-controlled measurements. The surface tension was measured at 50 °C to allow the DPPC particles to migrate to the newly formed air-liquid interfaces. A DPPC control (0.5 mg/mL) was also analyzed using the same procedure.

### Statistical analysis

All experiments were performed with three or more replicates unless stated. Statistical analysis was performed using JMP Pro 17 (SAS Institute Inc., Cary, NC). Direct comparisons of two cases utilized Students *t*-test with a significance limit of *p* = 0.05.

## Results

### Initial characterization of SLS-EEG formulations prior to storage

Spray drying of the feed dispersions to produce three batches of powder formulation resulted in a measured mean spray rate of ~0.40 ml/min with formulation bulk density of ~0.04 g/mL (Table 1). The recovered powder yields of the batches were between 70 and 77%. The moisture content of the powder batches and combined powder formulation was ≤1.5% w/w (Table 1). The measured DPPC content for the individual batches (~25% w/w) was very close to the nominal content (~99%). After combining all the batches together, the DPPC content (mean ± standard deviation) was 25.3 ± 1.7% w/w which is ~101.5% of the nominal content (Table 1).

**Figure 1.**
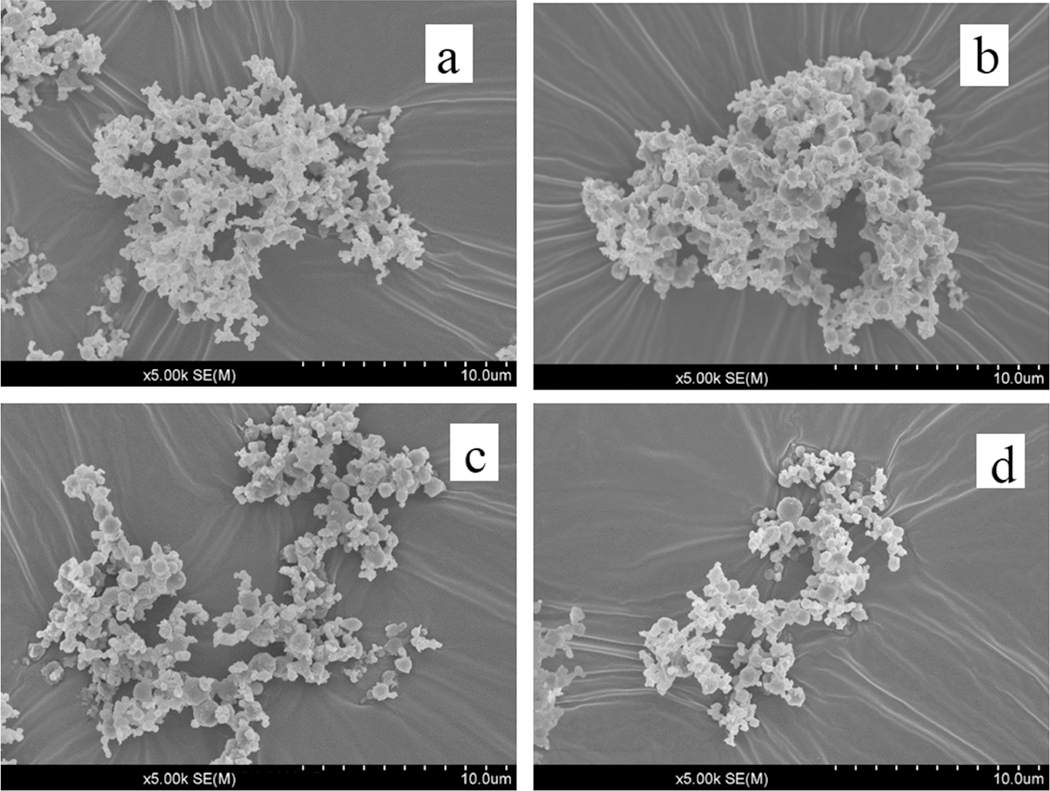
Scanning electron micrographs of spray-dried SLS-EEG powder formulation at (a) initial, (b) after 6 months storage at 25 ± 2 °C and 40 ± 5% RH, (c) after 6 months storage at 5 ± 2 °C and 40 ± 5% RH and (d) after 6 months storage at −20 ± 2 °C and 40 ± 5% RH.

Table 2 shows the particle size distribution of the spray-dried SLS-EEG powder formulations determined using Sympatec laser diffraction technique. The primary particle size (Dv50) of the individual powder batches and combined powder formulation determined at 4 bar dispersion pressure was ~0.9 μm with nearly 100% particles less than 5 μm. The scanning electron micrographs (Figure 1(a)) also support the micrometer sized powder particles. There was a large sub-micrometer fraction with 56% of particles being less than 1 μm, which is required for the EEG formulation delivery approach. At 0.5 bar, the Dv50 of the batches and combined powder formulation was ~1.4 μm (Table 2), suggesting good dispersibility of the powders. The percent coefficient of variation (%CV) for the Dv50 of the combined powder formulation at both 4 and 0.5 bar was ≤2.8 (Table 2).

**Figure 2.**
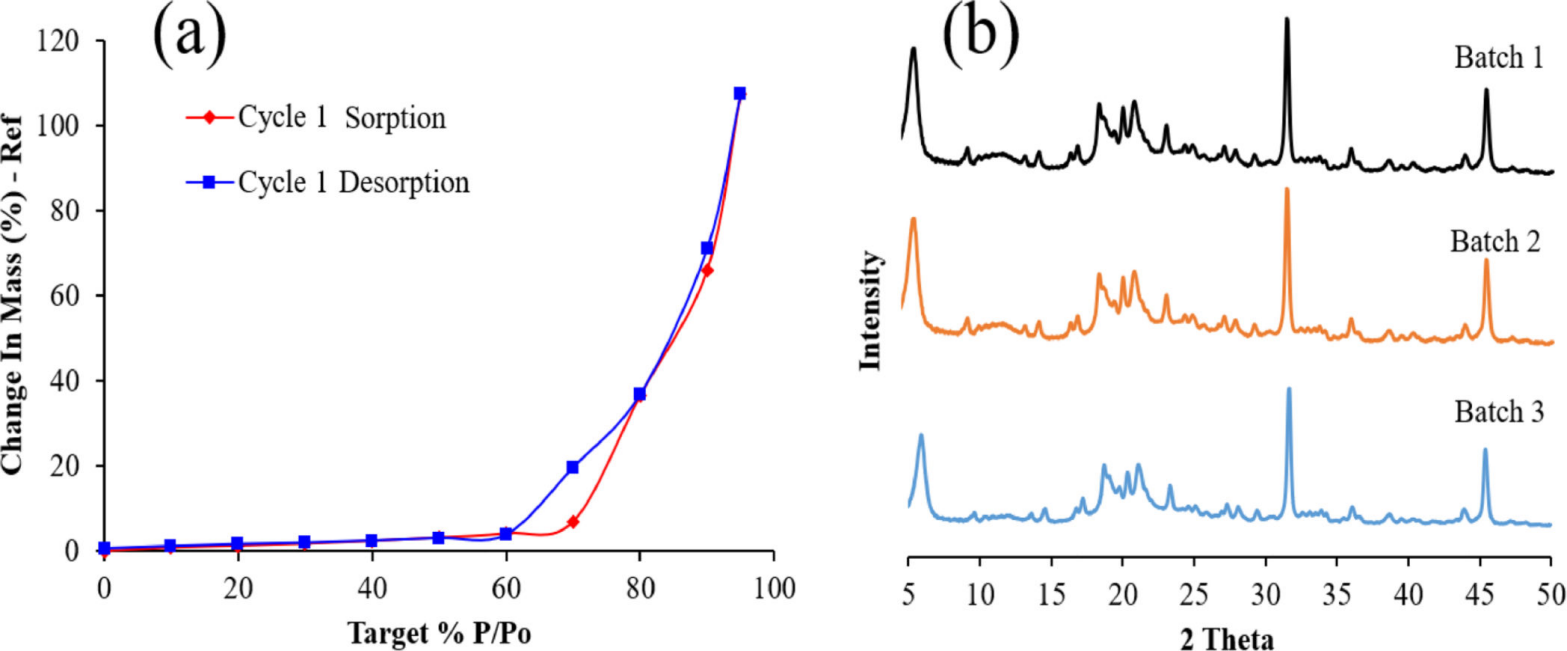
Dynamic vapor sorption behavior (a) and x-ray powder diffractograms (b) of spray-dried SLS-EEG powder formulation.

We also characterized the powder formulation for their moisture uptake and solid-state stability using DVS and XRPD, respectively. Figure 2(a) shows the moisture sorption behavior of the spray-dried SLS-EEG powder formulation. Following drying of the powder formulation during an equilibration period at 0% RH, less than 7% water uptake was observed by the powder from 10 to 70% RH (sorption), indicating relatively low uptake for an unprotected spray dried powder. Above 70% RH, significant moisture uptake was observed. The X-ray powder diffractograms (Figure 2(b)) of the spray-dried powder batches showed evidence of a partially crystalline structure. All the powders showed X-ray diffractogram peaks at the same positions as those for mannitol and l-leucine (not shown) with no obvious differences in the peak diffractograms among the batches indicating reproducible particle production process.

Aerosol performance and lung delivery efficiency of the SLS-EEG formulation using an infant air-jet DPI connected to a preterm nose-throat (NT) model was performed to establish the initial properties. The emitted dose of powder was >95% and the lung delivery efficiency (filter deposition) was ~50%, indicating high emptying of the device and good lung deposition.

### Storage stability of the SLS-EEG formulation

#### Physicochemical stability

Figure 1 shows the scanning electron micrographs of the spray-dried SLS-EEG powder formulation and shows a good agreement with the Sympatec laser diffraction size measurements with respect to the geometric diameter of the particles. The particles were spherical with wrinkled surfaces (Figure 1(a)). After 6 months storage at 5 ± 2 °C and 40 ± 5% RH and −20 ± 2 °C and 40 ± 5% RH, no obvious change in the particle morphology was observed (Figure 1(c,d)), however after storage at 25 ± 2 °C and 40 ± 5% RH more agglomerated particles were visible (Figure 1(b)).

The initial DPPC content of the powder was 25.3 ± 1.7% w/w which is 101 ± 6.7% of the nominal content (Table 3). After 3 months storage under the three temperature conditions (25 ± 2 °C, 5 ± 2 °C and −20 ± 2 °C), compared to the initial value, there were no significant differences observed in the DPPC content (23.8 ± 1.2% w/w at 25 °C, 23.7 ± 1.0% w/w at 5 °C and 24.0 ± 1.1% w/w at −20 °C, respectively) (Table 3). After 6 months storage at these conditions, the DPPC content remained similar to the initial values (Table 3).

Table 4 shows the particle size characteristics determined at dispersion pressures of 0.5 and 4.0 bar of the formulation after 3- and 6-months storage at three temperature conditions. The primary particle size of the micrometer sized formulation remained unchanged following 3- and 6-months storage at 5 and −20 °C (*p* > 0.05), with a small increase in primary particle size observed at 25 °C (*p* < 0.05) when measured at 4 bar. However, small changes (<3%) in the particle fractions (<1 μm and/or <5 μm) were observed during storage at all conditions, these small changes did not affect the formulation aerosol performance.

**Figure 3.**
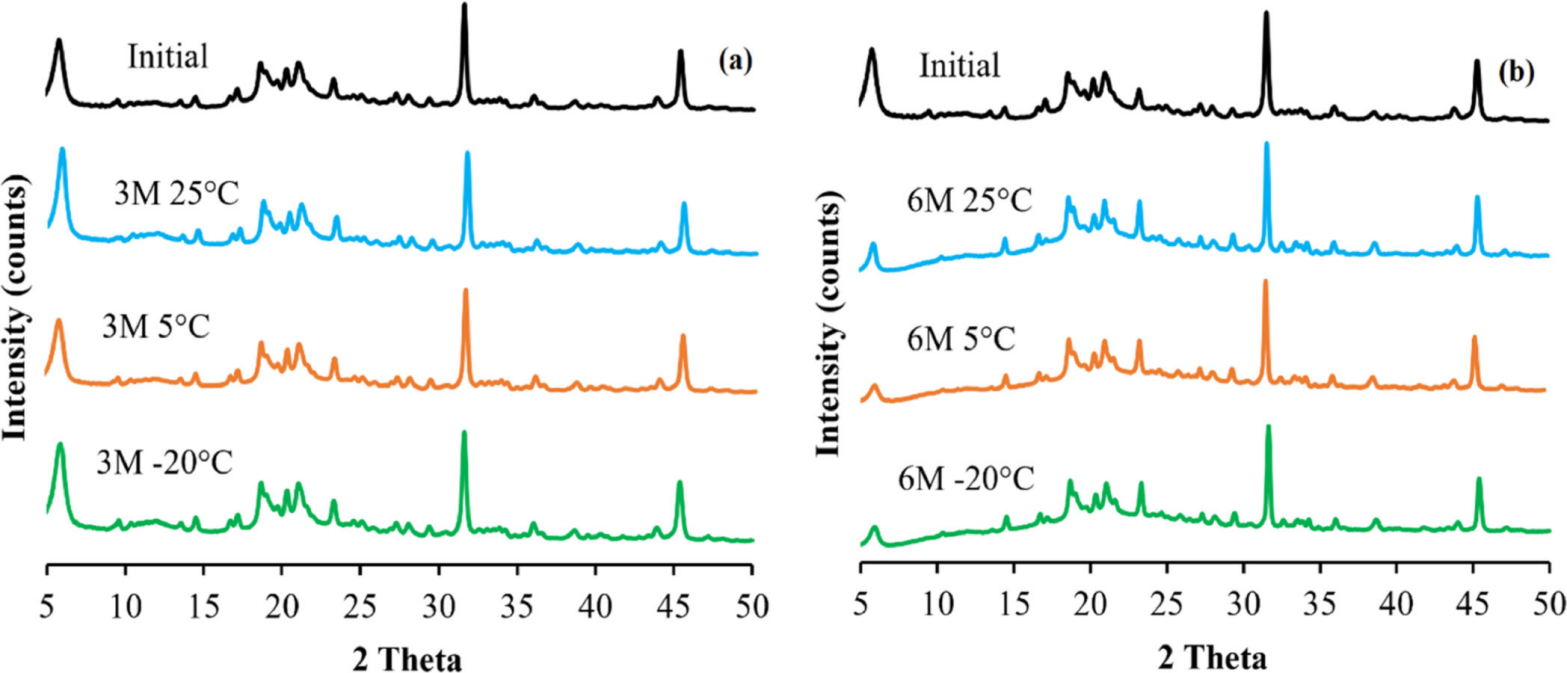
X-Ray diffractograms of spray-dried SLS-EEG powder formulation measured initially and after 3 months (3M) (a); initially and after 6 months (6 M) (b) storage at 25 ± 2 °C and 40 ± 5% RH, 5 ± 2 °C and 40 ± 5% RH and −20 ± 2 °C and 40 ± 5% RH.

The X-ray powder diffraction in Figure 3 showed evidence of crystalline structures in the SLS-EEG powder formulation initially and there were no obvious changes in the peak positions observed following 3 months (Figure 3(a)) and 6 months (Figure 3(b)) storage at the three temperature conditions suggesting no changes in the crystalline form during storage.

#### Aerosol performance stability

**Figure 4.**
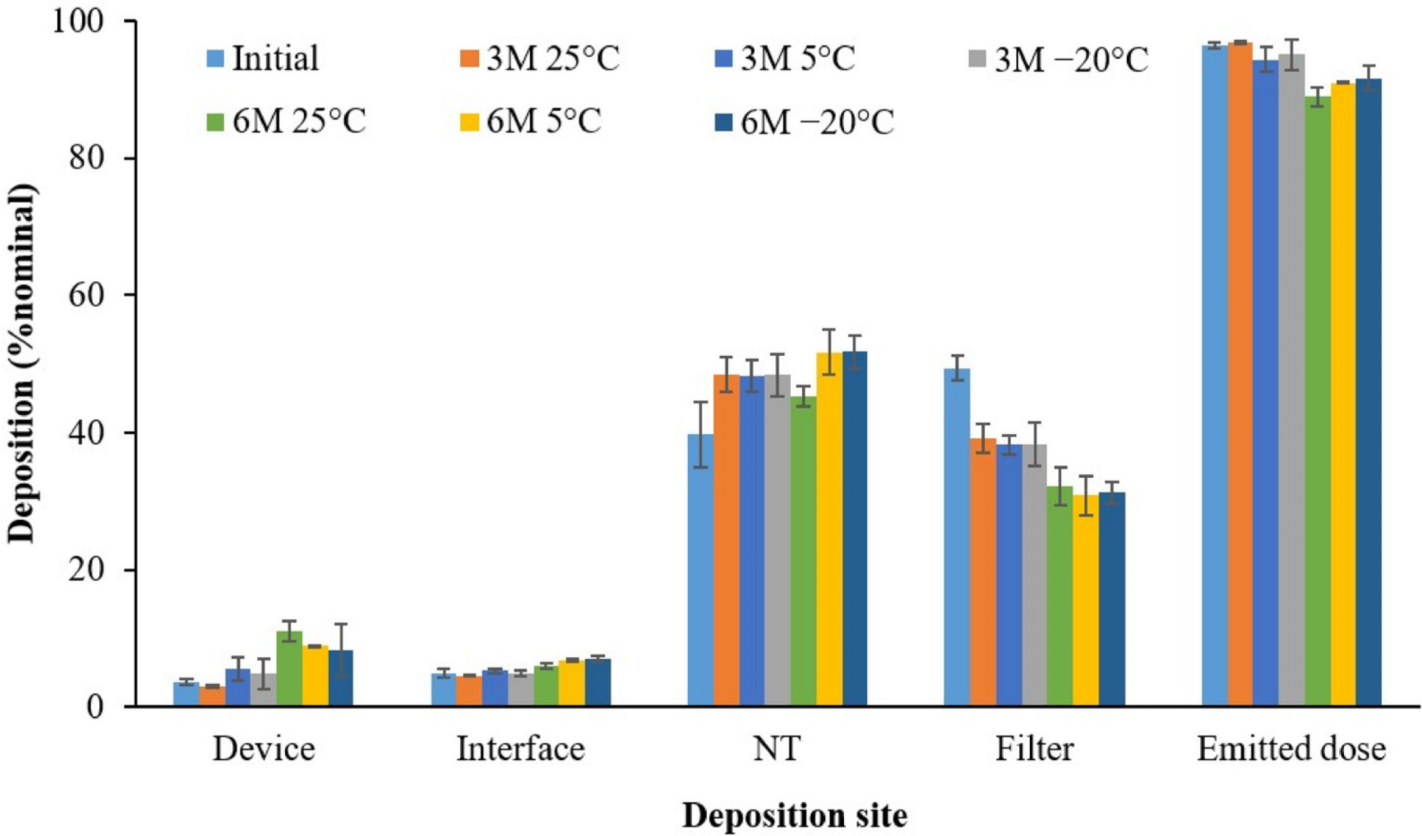
Aerosol performance of the spray-dried SLS-EEG powder formulation measured initially and after 3 and 6 months (3 M and 6 M) storage at 25 ± 2 °C and 40 ± 5% RH, 5 ± 2 °C and 40 ± 5% RH and −20 ± 2 °C and 40 ± 5% RH (bars represent the mean values; error bars represent standard deviations, *n* = 3; NT: nose-throat).

Figure 4 shows the *in vitro* determined regional deposition and emitted dose of the spray-dried SLS-EEG formulation following aerosolization from the D5 air-jet DPI connected to a preterm nose-throat (NT) model and operated at 4 LPM flow rate. The emitted dose of powder was 96.4 ± 0.4% initially and it remained unchanged (*p* > 0.05) during 3 months storage at the three temperature conditions (97.0 ± 0.2% at 25 °C, 94.5 ± 1.8% at 5 °C and 95.2 ± 2.2% at −20 °C, respectively). However, in comparison to initial performance, the lung delivery efficiency (% of filter deposition) was significantly lower (~40% vs ~50%) (*p* < 0.05) after 3 months storage under the three temperature conditions and it further decreased after 6 months storage under the three temperature conditions (~30% vs ~50%) (*p* < 0.05). The emitted dose was also significantly decreased (~90% vs ~96%) after 6 months storage under the three temperature conditions. There were no differences in the filter deposition (*p* > 0.05) among the three storage temperatures both after 3 (~40% filter deposition for all three storage temperatures) and 6 months storage conditions (~30% filter deposition for all three storage temperatures). On the other hand, in comparison to initial time point, for both 3- and 6-months storage conditions, the NT (nose-throat) deposition increased (~50% vs ~40%) (*p* < 0.05) which has contributed to decrease in the filter deposition during the storage.

#### In-use stability of the SLS-EEG formulation

**Figure 5.**
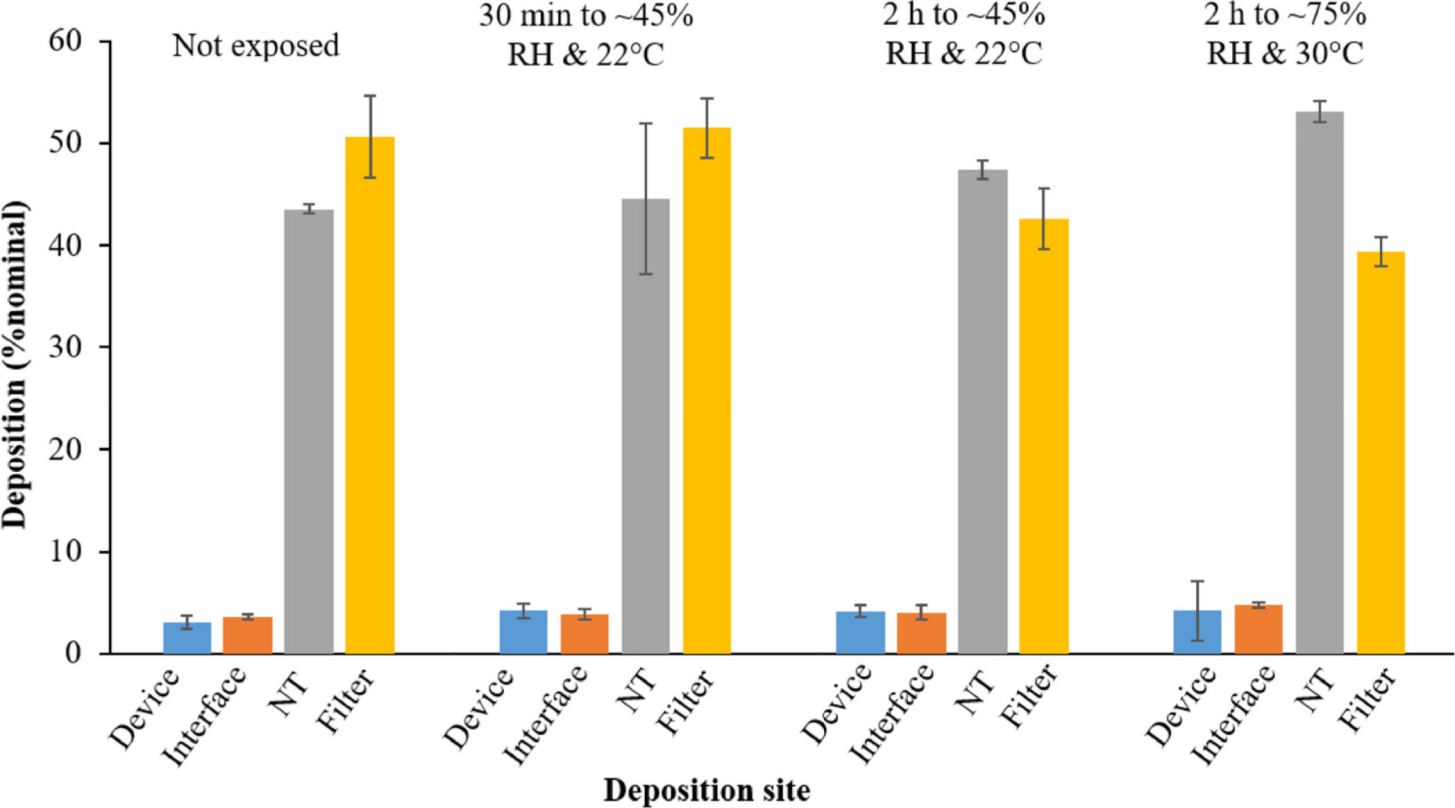
Aerosol performance of the spray-dried SLS-EEG powder formulation after in use exposure to 22 °C and 45% RH and 30 °C and 75% RH for 30 and 120 min (bars represent the mean values; error bars represent standard deviations, *n* ≤ 3; NT: nose-throat).

The in-use stability of the spray-dried SLS-EEG formulation was evaluated by aerosol performance testing after exposure of the formulation while loaded in the DPI to environmental conditions of 22 °C and 45% RH for 30 min and 120 min at 30 °C and 75% RH for 120 min. Figure 5 shows the data for in-use stability aerosol performance study. There was no difference in lung delivery efficiency (%filter deposition) following 30 min in device exposure to 22 °C and 45% RH (~50% nominal filter deposition) but increasing the exposure time to 120 min resulted a decrease in lung filter deposition (~40% of nominal filter deposition). Exposure to conditions of 30 °C and 75% RH for 120 min did not further reduce the lung delivery efficiency compared to 22 °C and 45% RH. For all the conditions, the DPI device retention remained similar (~4% of nominal) which resulted no change in the emitted dose (~96% of nominal) from the DPI device. These results suggest exposing the formulation when loaded in the inhaler to ambient humidity (up to 45% RH) did not affect either the emitted dose or lung dose if the inhaler was actuated within 30 min of device loading. Even at elevated temperature and humidity conditions (30 °C and 75% RH), formulation and device performance were not completely compromised with a similar emitted dose to the initial conditions and only a 10% reduction in the lung dose.

#### Surface activity of the SLS-EEG formulation

The dynamic surface tension of the powders was measured using bubble pressure tensiometer. Initially the mean ± SD surface tension of the spray-dried formulation measured at a bubble age of 100,000 ms was 31.5 ± 4.7 mN/m. The surface activity of the powders remained unchanged compared to the initial values with mean ± SD surface tension 29.4 ± 5.0 mN/m and 28.4 ± 4.5 mN/m following 3 months storage at 25 °C and 5 °C, respectively. Even after 6 months storage at 25 °C and 5 °C, the mean ± SD surface tension measured at a bubble age of 100,000 ms were 31.1 ± 4.9 and 33.3 ± 0.2, respectively suggesting the stable surface activity of the powders during 6 months storage.

## Discussion

In this study we evaluated the storage and in-use stability of a novel SLS-EEG spray-dried powder formulation that was composed of the phospholipid (DPPC), surfactant protein B peptide mimic (B-YL), hygroscopic excipients (mannitol and sodium chloride) and a dispersion enhancer (l-leucine). We produced three batches of the powder formulation and evaluated their physicochemical properties and aerosol performance. Initial evaluation for the DPPC content uniformity of individual batches and combined powder formulation (~100% of the nominal content, Table 1) suggested the capability of spray drying process to produce SLS-EEG powder without degradation of the DPPC.

The primary particle size (~1.0 μm) and the fine particle fraction <1 μm (56%) showed the suitability of the powder formulation for the EEG application. The particle size variability (%CV) of the individual batches and combined powder formulation (≤2.8%, Table 2), reflects the consistent size of the spray-dried batches and good reproducibility of the spray drying process. The moisture content (≤1.5% w/w) of the produced SLS-EEG powder formulation agrees with the previously reported results for the surfactant formulations [39]. The moisture uptake study showed low sensitivity of the unprotected spray-dried powder to water uptake up to 60% RH and the relatively hygroscopic nature of the powder in elevated humid environments (>70% RH) (Figure 2(a)). These properties are also favorable for the EEG formulation approach. The low water uptake at ambient conditions (10–60% RH), provides stability at typical storage or handling conditions for the formulation. The increased water uptake at >70% RH, provides hygroscopic growth in the humid lung environment which is necessary for particle growth and retention in the lung airways. The solid-state stability evaluated by X-ray powder diffraction technique showed no obvious changes in the peak positions after 3- and 6-months storage under all the storage conditions (Figure 3), suggesting the solid-state stability of the spray-dried formulation during 6-months storage. Walther et al. also reported the solid-state stability of different spray-dried surfactant powder formulations where no change in the crystallinity and peak positions were observed after 6 months storage at 40 °C and 75% relative humidity [39]. The particle morphology of the SLS-EEG powders also did not change during 6-months storage at 5 °C and −20 °C. However, agglomerated particles were visible after storage at 25 °C (Figure 1). Previously it has been reported that higher temperature facilitates particle agglomeration by overcoming electrostatic repulsion and promoting more inter-particle collisions [40,41]. Similar to the morphology, the primary particle size did not change after storage at 5 and −20 °C but changes were observed at 25 °C (Table 4). Changes in the particle fractions < 1 μm and < 5 μm were observed for all storage conditions with more prominent changes for the powder stored at 25 °C which could be due to the potential particle aggregation observed at this storage condition (Figure 1(b)) [42,43]. The small changes in the primary particle size may require that any future product be recommended for storage at <5 °C to maintain product quality and aerosol performance.

In comparison to initial values, the DPPC content did not change after 3- and 6-months storage at the three temperature conditions (Table 3), suggesting the chemical stability of DPPC in the formulation during 6-months storage at 25 °C and 40% RH, 5 °C and 40% RH and −20 °C and 40% RH.

Although the focus of this study was solely on assessing the stability of DPPC, we also evaluated the content of B-YL after 6 months storage at −20 ± 2 °C and 40 ± 5% RH. The B-YL content remained similar to initial (~52% of theoretical content) during the storage period. Preliminary investigations revealed that the Nano spray dryer, employing a mesh nebulizer, led to degradation of the B-YL protein mimic. These findings showed that the B-YL content in the SLS-EEG powder was ~56% of the nominal amount (5% w/w) [35], even though the nominal amount (5% w/w) of B-YL was present in the feed dispersions before and after completion of the spray drying. Notably, no loss in the B-YL content was observed when we prepared the same formulation using the nozzle-based Mini spray dryer, suggesting that the loss of B-YL is due to peptide degradation during the mesh-nebulizer-based Nano spray drying process [35]. Despite changes in the B-YL content following spray drying, we did observe *in vivo* activity of an SLS-EEG formulation produced using the Nano spray dryer [44], although we have now transitioned to the Mini nozzle-based dryer which is both scalable technology and does not alter the integrity of B-YL during spray drying. Walther et al. observed no degradation of B-YL peptide after 1-month storage for different spray-dried surfactant powder formulations at 5 °C [39].

Recently Momin et al. reported the physicochemical and aerosolization stability of spray-dried Survanta EEG powder formulation, where DPPC content also remained stable over 18 months storage at −20 °C and 0% RH [34]. The aerosol performance of the powder formulation following aerosolization from the D5 air-jet DPI connected to a preterm NT model showed high emptying (>95% emitted dose) of the device and good lung delivery efficiency (~50% filter deposition). The emitted dose did not change after 3-months storage at the three temperature conditions but it significantly decreased after 6-months storage for all the conditions which was due to increased powder retention in the DPI (~10% device after 6-months vs ~4% device initially and after 3-months). In comparison to initial performance, the lung delivery efficiency was also significantly lower (~40% vs ~50%) (*p* < 0.05) after 3-months storage under the three temperature conditions and it further decreased after 6-months storage (~30% vs ~50%) (*p* < 0.05). However, it remains to be determined if these small but significant changes in emitted dose and *in vitro* lung dose will affect the clinical efficacy of the product. The in-use stability of the SLS-EEG powder formulation showed no change in emitted dose and lung delivery efficiency following 30 min exposure when loaded in the device at 22 °C and 45% RH, suggesting that unprotected powder formulation could be loaded into the DPI device at ambient conditions (<45% RH) and actuated within 30 min of DPI loading without altering the device performance (emptying) and lung deposition. Even at elevated temperature and humidity conditions (30 °C and 75% RH), no change in the emitted dose compared to the initial conditions were observed and with only a 10% reduction in the lung dose. The surface tension measured using bubble pressure tensiometer showed stable surface activity of the powder formulations during 6-months storage at different temperature conditions which could be due to the chemical stability of the DPPC and B-YL during the storage period. It has been reported that over time there may be a chemical degradation of phospholipids and/or peptide which can lower the surface activities of surfactant formulations [45,46].

Although surfactant delivery as dry powder aerosol showed promising preliminary results in small human subject trial over several decades ago [8], the introduction of intratracheal administration of animal derived liquid lung surfactant in 1980s overshadowed the outcome of the preliminary dry powder surfactant delivery study [47] and became standard of care. However, due to the continuing challenges associated with the intratracheal delivery of liquid surfactant and respiratory related side effects arising from pouring a high liquid volume down an endotracheal tube, and the requirement of a relatively high dosage of surfactant, a viable alternative delivery method may be a surfactant dry powder aerosol which could eliminate these problems and could be delivered without the need for invasive intubation. Recently Walther et al. reported the efficacy of the dry powder synthetic lung surfactant formulations in surfactant-deficient rabbits and preterm lambs administered in combination with noninvasive respiratory support [15,48]. This study showed comparable efficacy (arterial oxygenation and lung compliance) of dry powder synthetic lung surfactant powder formulation to the commercial liquid surfactant formulation [48]. Recently we reported the development and *in vivo* efficacy of a novel synthetic lung surfactant EEG (SLS-EEG) powder formulation delivered as an aerosol through an intratracheal interface [35,44]. In this study, we observed similar arterial oxygenation for the liquid-instilled surfactant and the dry powder aerosol groups but superior lung compliance in powder aerosol group at *a* ~10-fold lower phospholipid dose of dry powder aerosol group (24 mg/kg vs 200 mg/kg bodyweight of phospholipids for aerosol and liquid group, respectively) [44]. Since the ultimate purpose of this project is to develop a cost-effective aerosol formulation and delivery device combination to be able to use in a low- and middle-income countries (LMIC) we are continuing to optimize the formulations composition. The first generation SLS-EEG formulation reported in this study is physico-chemically stable and showed good aerosol performance.

Our overall goal is to produce a DPI device/formulation combination to be used in low resource settings by healthcare providers with minimal training, by simply loading the powder formulation, inserting the cannula into the infant’s nostrils and actuating the inhaler. Development of a micrometer sized aerosol and use of the EEG technology will minimize exhalation losses. Along with the formulation optimization we are also continuing to optimize the device to make it user friendly for the LMIC environment. Although the device-formulation combination reported in this study showed promising results and has the potential to be used with neonates to treat RDS, there are some potential challenges that could be observed when delivering dry powders to neonates using our air-jet device, especially given the unique physiological and clinical considerations of the patient population. The narrow, short, and highly dynamic airways of neonates increase the risk of upper airway deposition and reduces the lower lung deposition, lowering therapeutic efficacy. The variability in airflow and patient cooperation could cause dose variability from one administration to another however this can be overcome using the positive pressure administration. Moreover, neonatal respiratory support often involves humidified air which can affect powder flow properties and agglomerate particles, reducing aerosol quality and drug delivery efficiency. Furthermore, although our aim is to deliver a micrometer sized aerosol through the nasal passages, depositing of particles can potentially irritate delicate neonatal respiratory tissues which can induce coughing, bronchospasm, or mucosal irritation. We have not observed any visible occlusion of the *in vitro* airways and our *in vivo* studies have successfully delivered 30 mg doses through a 3.5 mm endotracheal tube demonstrating feasibility of the proposed approach.

## Conclusions

We have successfully produced and characterized the physicochemical and aerosolization stability of an SLS-EEG powder formulation. Solid-state stability and surface activity remained unchanged following storage for 6 months at 25 °C, 5 °C and −20 °C, with only small changes in the primary particle size observed following storage at 25 °C. The *in vitro* lung delivery efficiency was high for dry powder delivery to a simulated neonatal airway. A decreased lung dose from ~50% to ~40% was observed following 3 months storage at 25 °C, 5 °C and −20 °C, however this remained acceptable for this challenging delivery route. In-use testing also indicated that some small changes in delivery efficiency were observed under extreme environmental conditions but a high lung dose (~40%) was delivered despite exposure to 75% RH and 30 °C for 120 min.

## Figures and Tables

**Table 1.** Spray rate, bulk density, DPPC content and moisture content of three different batches of the spray-dried SLS-EEG formulations (for DPPC content, data are mean ± standard deviation, *n* = 3, for spray rate, bulk density and moisture content, *n* = 1).

Powder batch	Spray rate (mL/min)	Bulk density (g/mL)	DPPC content		Moisture content
			% w/w	% Recovery	% w/w

Batch 1	0.44	0.04	24.8 ± 1.3	99.4 ± 5.2	1.2
Batch 2	0.42	0.04	24.8 ± 0.8	99.5 ± 3.2	1.5
Batch 3	0.42	0.04	24.7 ± 0.9	99.1 ± 3.7	1.3
Combined	–	–	25.3 ± 1.7	101.4 ± 6.7	1.3

**Table 2.** Primary particle size of three different batches and the combination of three batches of the spray-dried SLS-EEG powder formulation determined by sympatec laser diffraction method (data are for a single measurement for each batch at each dispersion pressure, for combined powder data are mean ± standard deviation, *n* = 3, data in the parenthesis are the %CV).

	Primary particle size, Dv50 (μm)		Particle fraction (%)			
			<1 μm		<5 μm	
Powder batch	0.5 bar	4 bar	0.5 bar	4 bar	0.5 bar	4 bar

Batch 1	1.4	0.9	32	56	96	100
Batch 2	1.4	0.9	33	56	97	100
Batch 3	1.4	1.0	32	54	96	100
Combined	1.4 ± 0.0 (2.8%)	0.9 ± 0.0 (2.2%)	33 ± 0.9 (2.6%)	56 ± 1.9 (3.4%)	97 ± 0.5 (0.6%)	100 ± 0.0 (0.0%)

**Table 3.** DPPC content of the spray-dried SLS-EEG powder formulations measured initially and after 3 and 6 months (3 M and 6 M) storage at 25 ± 2 °C and 40 ± 5% RH, 5 ± 2 °C and 40 ± 5% RH and −20 ± 2 °C and 40 ± 5% RH (data are mean ± standard deviation, *n* = 3).

	DPPC content	
Storage	%w/w	% Recovery

Initial	25.3 ± 1.7	101 ± 6.7
3M 25 °C	23.8 ± 1.2	96 ± 5.0
3M 5 °C	23.7 ± 1.0	95 ± 3.9
3M −20 °C	24.0 ± 1.1	96 ± 4.3
6M 25 °C	24.4 ± 0.3	98 ± 1.3
6M 5 °C	24.6 ± 0.2	99 ± 0.7
6M −20 °C	24.2 ± 0.7	97 ± 2.9

* Significant difference compared to initial; Student’s *t*-test, *p* < 0.05.

**Table 4.** Primary particle size of the spray-dried SLS-EEG powder formulation measured initially and after 3 and 6 months (3 M and 6 M) storage at 25 ± 2 °C and 40 ± 5% RH, 5 ± 2 °C and 40 ± 5% RH and −20 ± 2 °C and 40 ± 5% RH (data are mean ± standard deviation, *n* = 3).

	Primary particle size, DV50 (μm)		Particle fraction (%)			
			<1 μm		<5 μm	
Storage	0.5 bar	4 bar	0.5 bar	4 bar	0.5 bar	4 bar

Initial	1.4 ± 0.0	0.9 ± 0.0	33 ± 0.9	56 ± 1.9	97 ± 0.6	100 ± 0.0
3M 25 °C	1.7 ± 0.0*	1.0 ± 0.0*	27 ± 0.5*	51 ± 0.6*	94 ± 0.5*	100 ± 0.3
3M 5 °C	1.4 ± 0.1	1.0 ± 0.0	33 ± 1.2	53 ± 0.5*	97 ± 1.5	100 ± 0.0
3M −20 °C	1.5 ± 0.0	0.9 ± 0.0	31 ± 0.8*	55 ± 1.2	94 ± 0.7*	100 ± 0.0
6M 25 °C	1.9 ± 0.0*	1.0 ± 0.0*	22 ± 0.0*	49 ± 0.1*	89 ± 0.5*	97 ± 0.0*
6M 5 °C	1.4 ± 0.0	0.9 ± 0.0	33 ± 1.3	57 ± 0.0	97 ± 0.2	100 ± 0.0
6M −20 °C	1.5 ± 0.0	0.9 ± 0.0	30 ± 0.6*	56 ± 0.1	95 ± 0.4*	100 ± 0.0

* Significant difference; Student’s *t*-test, *p* < 0.05.

## Data Availability

Supporting data are openly available in OSF: https://osf.io/yt8ec/?view_only=c7c573ab302c488787ab941c68d01cc3.

## References

[1] Sardesai S, Biniwale M, Wertheimer F, Evolution of surfactant therapy for respiratory distress syndrome: past, present, and future. Pediatr Res. 2017;81(1–2):240–248. doi:10.1038/pr.2016.203. 27706130

[2] Dyer J Neonatal respiratory distress syndrome: tackling a worldwide problem. Pharm Ther. 2019;44:12. PMC6336202 30675087

[3] El-Gendy N, Kaviratna A, Berkland C, Delivery and performance of surfactant replacement therapies to treat pulmonary disorders. Ther Deliv. 2013;4(8):951–980. doi:10.4155/tde.13.72. 23919474 PMC3840129

[4] Shah S Exogenous surfactant: intubated present, nebulized future? World J Pediatr. 2011;7(1):11–15. doi:10.1007/s12519-010-0201-4. 20549420

[5] Gupta S, Donn SM. Novel approaches to surfactant administration. Crit Care Res Pract. 2012;2012:278483. doi:10.1155/2012/278483. PMC3518953 23243504

[6] Willson DF. Aerosolized surfactants, anti-inflammatory drugs, and analgesics. Respir Care. 2015;60(6):774–793. doi:10.4187/respcare.03579. 26070574

[7] Willson DF, Notter RH. The future of exogenous surfactant therapy. Respir Care. 2011;56(9):1369–1388. doi:10.4187/respcare.01306. 21944686

[8] Morley CJ, Miller CW, Bangham AD, Dry artificial lung surfactant and its effect on very premature babies. Lancet. 1981;1(8211):64–68. doi:10.1016/s0140-6736(81)90002-7. 6109119

[9] Walther FJ, Waring AJ. Aerosol delivery of lung surfactant and nasal CPAP in the treatment of neonatal respiratory distress syndrome. Front Pediatr. 2022;10:923010. doi:10.3389/fped.2022.923010. PMC9240419 35783301

[10] Bianco F, Ricci F, Catozzi C, From bench to bedside: in vitro and in vivo evaluation of a neonate-focused nebulized surfactant delivery strategy. Respir Res. 2019;20:134. 31266508 10.1186/s12931-019-1096-9 PMC6604359

[11] Sood BG, Cortez J, Kolli M, Aerosolized surfactant in neonatal respiratory distress syndrome: phase I study. Early Hum Dev. 2019;134:19–25. doi:10.1016/j.earlhumdev.2019.05.005. 31121339

[12] Berggren E, Liljedahl M, Winbladh B, Pilot study of nebulized surfactant therapy for neonatal respiratory distress syndrome. Acta Paediatr. 2000;89(4):460–464. doi:10.1111/j.1651-2227.2000.tb00084.x. 10830460

[13] Finer NN, Merritt TA, Bernstein G, An open label, pilot study of Aerosurf^®^ combined with nCPAP to prevent RDS in preterm neonates. J Aerosol Med Pulm Drug Deliv. 2010;23(5):303–309. doi:10.1089/jamp.2009.0758. 20455772

[14] Pillow JJ, Minocchieri S. Innovation in surfactant therapy II: surfactant administration by aerosolization. Neonatology 2012;101(4):337–344. doi:10.1159/000337354. 22940623

[15] Walther FJ, Hernández-Juviel JM, Waring AJ. Aerosol delivery of synthetic lung surfactant. PeerJ. 2014;2:e403. doi:10.7717/peerj.403. 24918030 PMC4045332

[16] Samsudin DD. Current issues and challenges in the use of aerosolized surfactant for respiratory distress syndrome in the newborns. Indones Biomed J. 2013;5(2):91–100. doi:10.18585/inabj.v5i2.57.

[17] LiCalsi C, Christensen T, Bennett JV, Dry powder inhalation as a potential delivery method for vaccines. Vaccine 1999;17(13–14):1796–1803. doi:10.1016/s0264-410x(98)00438-1. 10194842

[18] Ibrahim M, Verma R, Garcia-Contreras L. Inhalation drug delivery devices: technology update. Med Devices (Auckl). 2015;8:131–139. doi:10.2147/MDER.S48888. 25709510 PMC4334339

[19] Islam N, Cleary MJ. Developing an efficient and reliable dry powder inhaler for pulmonary drug delivery—a review for multidisciplinary researchers. Med Eng Phys. 2012;34(4):409–427. doi:10.1016/j.medengphy.2011.12.025. 22277307

[20] Weers JG, Son Y-J, Glusker M, Idealhalers versus realhalers: is it possible to bypass deposition in the upper respiratory tract? J Aerosol Med Pulm Drug Deliv. 2019;32(2):55–69. doi:10.1089/jamp.2018.1497. 30481087

[21] Moon C, Smyth HD, Watts AB, Delivery technologies for orally inhaled products: an update. AAPS PharmSciTech. 2019;20(3):117. doi:10.1208/s12249-019-1314-2. 30783904

[22] Newman SP, Busse WW. Evolution of dry powder inhaler design, formulation, and performance. Respir Med. 2002; 96(5):293–304. doi:10.1053/rmed.2001.1276. 12113378

[23] Walsh BK, DiBlasi RM. Mechanical ventilation of the neonate and pediatric patient. In: Walsh BK, Czervinske MP, DiBlasi RM, editors. Perinatal and Pediatric Respiratory Care. Saunders Elsevier; 2010. p. 325–347.

[24] Worth Longest P, Hindle M. Numerical model to characterize the size increase of combination drug and hygroscopic excipient nanoparticle aerosols. Aerosol Sci Technol. 2011;45(7):884–899. doi:10.1080/02786826.2011.566592. 21804683 PMC3143486

[25] Howe C, Hindle M, Bonasera S, Initial development of an air-jet dry powder inhaler for rapid delivery of pharmaceutical aerosols to infants. J Aerosol Med Pulm Drug Deliv. 2021;34(1):57–70. doi:10.1089/jamp.2020.1604. 32758026 PMC8182481

[26] Longest W, Farkas D, Bass K, Use of computational fluid dynamics (CFD) dispersion parameters in the development of a new DPI actuated with low air volumes. Pharm Res. 2019;36(8):110. doi:10.1007/s11095-019-2644-1. 31139939 PMC7324281

[27] Bass K, Farkas D, Longest W. Optimizing aerosolization using computational fluid dynamics in a pediatric air-jet dry powder inhaler. AAPS PharmSciTech. 2019;20(8):329. doi:10.1208/s12249-019-1535-4. 31676991 PMC7324282

[28] Boc S, Momin MAM, Farkas DR, Development and characterization of excipient enhanced growth (EEG) surfactant powder formulations for treating neonatal respiratory distress syndrome. AAPS PharmSciTech. 2021;22(4):136. doi:10.1208/s12249-021-02001-1. 33860409 PMC8274457

[29] Boc S, Momin MAM, Farkas DR, Performance of low air volume dry powder inhalers (LV-DPI) when aerosolizing excipient enhanced growth (EEG) surfactant powder formulations. AAPS PharmSciTech. 2021;22(4):135. doi:10.1208/s12249-021-01998-9. 33860378 PMC8268434

[30] Momin MAM, Howe C, Longest W, Surfactant powder delivery from infant air-jet dry powder inhaler (DPI): effect of flow rate and DPI design. Respiratory drug delivery (RDD) 2022 conference; 2022; Omni Orlando, ChampionsGate, Florida, USA held on May 1–5.

[31] Cal K, Sollohub K. Spray drying technique. I: hardware and process parameters. J Pharm Sci. 2010;99(2):575–586. doi:10.1002/jps.21886. 19774644

[32] Vehring R Pharmaceutical particle engineering via spray drying. Pharm Res. 2008;25(5):999–1022. doi:10.1007/s11095-007-9475-1. 18040761 PMC2292490

[33] Chang RYK, Chen L, Chen D, Overcoming challenges for development of amorphous powders for inhalation. Expert Opin Drug Deliv. 2020;17(11):1583–1595. doi:10.1080/17425247.2020.1813105. 32811193

[34] Momin MAM, Farkas D, Longest W, Long-term storage stability of an excipient enhanced growth (EEG) pulmonary surfactant powder formulation. Respiratory drug delivery (RDD) 2022 conference; 2022; Omni Orlando, ChampionsGate, Florida, USA held on May 1–5, 2022.

[35] Momin MAM, Farkas D, Hindle M, Development of a new dry powder aerosol synthetic lung surfactant product for neonatal respiratory distress syndrome (RDS) – Part I: in vitro testing and characterization. Pharm Res. 2024;41(8):1703–1723. doi:10.1007/s11095-024-03760-9. 39112775 PMC11362531

[36] Momin MAM, Howe C, Longest W, Physicochemical and aerosolization performance stability of an excipient enhanced growth (EEG) synthetic lung surfactant powder formulation. Drug delivery to the lungs (DDL) conference proceedings 2022 at Edinburgh International Conference Centre, Edinburgh, UK held on December 7–9; 2022; Vol. 33. p. 122–125.

[37] Howe C, Momin MAM, Farkas DR, Advancement of the infant air-jet dry powder inhaler (DPI): evaluation of different positive-pressure air sources and flow rates. Pharm Res. 2021;38(9):1615–1632. doi:10.1007/s11095-021-03094-w. 34462876 PMC8642819

[38] Li D, Xiong X, Bai Q, Development and validation of an LC–MS/MS method for quantification of dipalmitoylphosphatidylcholine as a promising biomarker for renal failure in urine. J Chin Pharm Sci. 2015;24(2):73. doi:10.5246/jcps.2015.02.008.

[39] Walther FJ, Chan H, Smith JR, Aerosol, chemical and physical properties of dry powder synthetic lung surfactant for noninvasive treatment of neonatal respiratory distress syndrome. Sci Rep. 2021;11(1):16439. doi:10.1038/s41598-021-95999-0. 34385559 PMC8360972

[40] Wang L, Yang X, Wang Q, Effects of ionic strength and temperature on the aggregation and deposition of multi-walled carbon nanotubes. J Environ Sci (China). 2017;51:248–255. doi:10.1016/j.jes.2016.07.003. 28115136

[41] Simmons MJ, Jayaraman P, Fryer PJ. The effect of temperature and shear rate upon the aggregation of whey protein and its implications for milk fouling. J. Food Eng. 2007;79(2):517–528. doi:10.1016/j.jfoodeng.2006.02.013.

[42] Andersson IM, Bergenståhl B, Millqvist-Fureby A, Particle morphology and rehydration properties of spray-dried microgels and fractal aggregates with varying fractions of native milk serum proteins. Int Dairy J. 2021;112:104862. doi:10.1016/j.idairyj.2020.104862.

[43] Kwon YB, Kang JH, Han CS, The effect of particle size and surface roughness of spray-dried bosentan microparticles on aerodynamic performance for dry powder inhalation. Pharmaceutics. 2020;12(8):765. doi:10.3390/pharmaceutics12080765. 32823545 PMC7465523

[44] DiBlasi RM, KenKnight H, Kontoudios N, Development of a new dry powder aerosol synthetic lung surfactant product for neonatal respiratory distress syndrome (RDS)–Part II: in vivo efficacy testing in a rabbit surfactant washout model. Pharm Res. 2024;41(9):1827–1842. doi:10.1007/s11095-024-03754-7. 39237797 PMC11436456

[45] Waring AJ, Gupta M, Gordon LM, Stability of an amphipathic helix-hairpin surfactant peptide in liposomes. Biochim Biophys Acta. 2016;1858(12):3113–3119. doi:10.1016/j.bbamem.2016.09.014. 27664499 PMC5096961

[46] Walther FJ, Gupta M, Gordon LM, A sulfur-free peptide mimic of surfactant protein B (B-YL) exhibits high in vitro and in vivo surface activities. Gates Open Res. 2018;2:13. doi:10.12688/gatesopenres.12799.2. 30234192 PMC6139377

[47] Walther FJ, Gordon LM, Waring AJ. Advances in synthetic lung surfactant protein technology. Expert Rev Respir Med. 2019;13(6):499–501. doi:10.1080/17476348.2019.1589372. 30817233

[48] Walther FJ, Gupta M, Lipp MM, Aerosol delivery of dry powder synthetic lung surfactant to surfactant-deficient rabbits and preterm lambs on non-invasive respiratory support. Gates Open Res. 2019;3:6. doi:10.12688/gatesopenres.12899.2. 31131369 PMC6480449

